# A Systematic Review and Integrated Bioinformatic Analysis of Candidate Genes and Pathways in the Endometrium of Patients With Polycystic Ovary Syndrome During the Implantation Window

**DOI:** 10.3389/fendo.2022.900767

**Published:** 2022-07-01

**Authors:** Zulazmi Sutaji, Marjanu Hikmah Elias, Mohd Faizal Ahmad, Abdul Kadir Abdul Karim, Muhammad Azrai Abu

**Affiliations:** ^1^ Department of Obstetrics & Gynecology, Faculty of Medicine, Universiti Kebangsaan Malaysia, Kuala Lumpur, Malaysia; ^2^ Faculty of Medicine & Health Sciences, Universiti Sains Islam Malaysia, Bandar Baru Nilai, Malaysia

**Keywords:** polycystic ovary syndrome, implantation window, endometrium, differentially expressed genes, systematic review

## Abstract

Polycystic ovary syndrome (PCOS) is a common disorder with wide-ranging clinical heterogeneity that causes infertility. However, the comprehensive molecular mechanisms of PCOS in causing infertility is remaining unclear. Hence, a comprehensive literature search was conducted using PubMed, Scopus, EBSCOhost, and Science Direct. Medical Subject Heading (MeSH) terms like PCOS, gene expression, implantation window and endometrium were used as the keywords. From 138 studies retrieved, original articles with RNA profiling on human endometrial tissues in PCOS women during the implantation window were included. Study design, sample size, sample type, method, and differentially expressed genes (DEGs) were identified from all publications. The DEGs were analyzed using the software packages DAVID, STRING, and Cytoscape. Three studies that met inclusion criteria were included, and 368 DEGs were identified. Twelve significant clusters from the protein-protein interaction network (PPI) complex were found, and cluster 1 showed very high intermolecular interactions. Five candidate genes (AURKA, CDC25C, KIF23, KIF2C, and NDC80) were identified from the systematic review and integrated bioinformatics analysis. It is concluded that cell cycle is the fundamental biological processes that were dysregulated in the endometrium of PCOS women, affecting decidualization progression in the endometrium during the implantation window.

## Introduction

Polycystic ovary syndrome (PCOS) is a leading cause of female infertility. It is characterized by anovulation, hyperandrogenism, and polycystic-appearing ovaries. PCOS prevalence varies among countries worldwide from 2.2% to 20% depending on the population studied and criteria used for diagnosis (1). PCOS is the most common endocrine disorder in reproductive-age women, and it is also one of the most extensively studied diseases. Nevertheless, many questions regarding its pathogenesis, diagnosis, clinical manifestations, complications, and treatment remain unanswered (2).

PCOS is a multifactorial disease that is caused by reproductive and metabolic endocrinopathy. As a multifactorial disease, development of PCOS involves complex pathophysiology and molecular pathogenesis (2, 3). The mechanisms involved in PCOS development impair the ovulation process and cause infertility among PCOS women. Many studies have reported on the correlation between ovulation and pregnancy rates among PCOS women (4, 5). However, apart from ovulation, appropriate priming of the endometrium *via* decidualization during implantation window together with embryo quality also contributes to successful implantation (6). During the implantation window, progesterone promotes decidualization process of endometrial stromal cells, leading to the changes of stromal cells’ cytoskeleton and optimal environment of cytokines and immunomodulators for blastocyst implantation (7). The changes in cytoskeleton help the endometrium to prepare for implantation by regulating trophoblast invasion (7). This process of blastocyte implantation occurs only during the implantation window. Thus, investigating the molecular mechanism in the endometrium during implantation window is essential to solve the infertility problem among PCOS women.

In PCOS women, the growth and differentiation of endometrium are largely influenced by androgens, insulin, and estrogen, but not progesterone (7). Thus, resulting in the absence of ovulation due to the absent of secretory transformation as the endometrium is constantly exposed to the stimulating and mitogenic effects of estradiol (7). The constant exposure of endometrium to estradiol may lead to endometrium overgrowth, unpredictable bleeding patterns, hyperplasia and cancer (7).

Generally, physiological changes during implantation window is describe based on specific anatomical and functional changes in the glands, blood vessels and endometrial stroma (7). However, the comprehensive molecular mechanisms of PCOS still remain unclear as many studies on PCOS have focused on a single cohort or a single molecular pathway. Microarray gene-expression profiling determines the expression pattern of many genes simultaneously at the transcription level (8). Microarray gene-expression profiling has emerged as an efficient technique for cancer diagnosis, prognosis, and molecular-pathway determination (9). In recent years, many studies on microarray gene-expression profiling have been conducted on PCOS, and hundreds of DEGs have been identified (10–13). However, the data generated are from a single patient cohort with a limited sample number. Thus, an integrated bioinformatics analysis that combines data from various cohorts could overcome this disadvantage. Identifying DEGs and enriching their biological functions and key pathways can improve our understanding of the molecular mechanisms that occur in the endometrium of PCOS women, especially during the implantation window.

## Materials and Methods

### Search Strategy

The systematic review was conducted according to the consensus PRISMA. A comprehensive data search was conducted using PubMed, Scopus, EBSCOhost, and Science Direct to identify related research publications with an unlimited starting publication date until October 1, 2021. The term “implantation window” and the Medical Subject Heading (MeSH) terms such as “polycystic ovary syndrome”, “gene expression” and “endometrium” were used as the keywords in the all field. The search strategy involved a combination (“AND”) of the following sets of keywords (1): “polycystic ovary syndrome” OR “polycystic ovarian syndrome” OR “PCOS,” (2) “endometrium” OR “endometrial,” (3) “implantation window,” and (4) “gene expression”. The term “gene expression” has been removed during the data search using PubMed, due to the low number of recovered papers. Synonyms for keywords were generated through MeSH terms from the Cochrane Library. Additional text terms were found by assessing collected review articles. Additional references were identified from the bibliographies of the retrieved studies.

### Inclusion Criteria

Case-control and prospective observational studies with abstracts investigating the DEGs in the endometrium of PCOS women during implantation window were included. Only clinical studies that used microarray method to identify DEGs in PCOS women were included to assure the homogeneity of the data. Other gene expression method such as quantitative PCR was not selected to avoid bias in gene selection.

### Exclusion Criteria

Publications with no primary data such as editorials, case reports, conference proceedings, and narrative review articles were excluded. *In silico*, *In vitro*, and *In vivo* studies were also excluded. This review focused on DEGs in the endometrium of PCOS women during the implantation window. Therefore, studies involving responses toward any treatment or intervention studies on a new treatment for PCOS were excluded. Studies that used ovary tissue biopsy, blood, and tissues other than endometrium as their sample were excluded. These assortment criteria were used to achieve the objective of this systematic review in determining the typical gene-expression signature in PCOS women and the related pathways that could be involved in PCOS development.

### Screening of Articles for Eligibility

**Figure 1:**
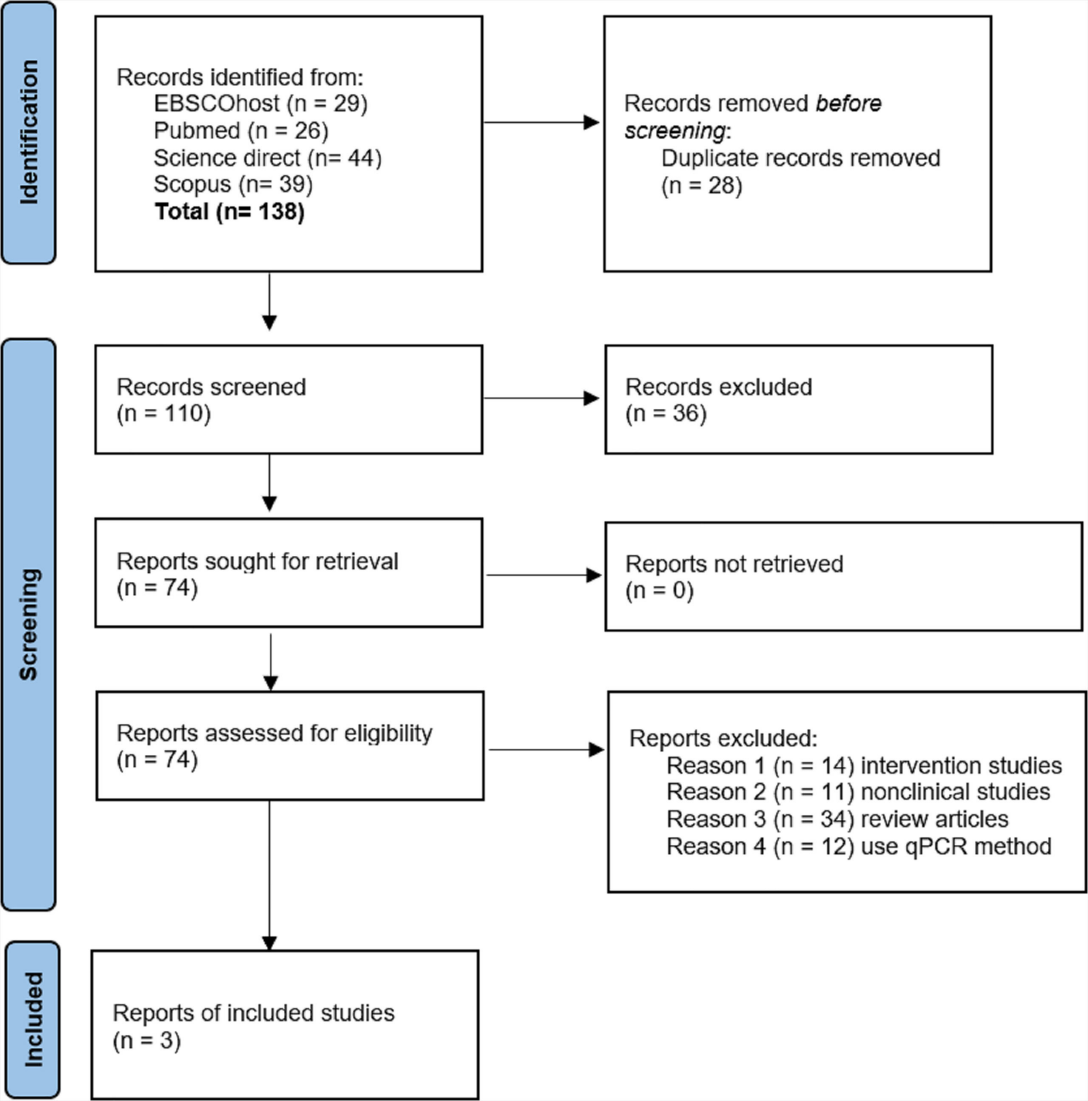
PRISMA flow diagram for studies selection in this systematic review.

Articles recovered from all resources were screened in three phases. All articles with titles that did not match the inclusion criteria were excluded, and duplicates were removed in the first phase. The abstracts of the remaining articles were examined, and articles that did not meet the inclusion criteria were excluded in the second phase. Finally, the full texts of the remaining articles were read and reviewed thoroughly. Systematic reviews, meta-analyses, *in vitro*, *in vivo*, and in silico, and articles that did not meet the inclusion criteria were excluded in this third phase. All the authors were involved in the screening, selection, and data extraction phase. **Figure 1** shows the flow chart summarizing the article assortment process and the reasons for article elimination.

### Data Extraction

All the authors were involved in the data extraction phase. Any discrepancies in opinions were decided by discussion between the authors. All data extraction was conducted independently using a data collection form to standardize the data collection, and records on reasons for rejection were kept. Data were extracted from the studies that fulfilled the inclusion criteria. Data collected from these studies included the following (1): title and author’s name (2), study design (3), objective (4), sample size (5), type of sample collected (6), method used in gene-expression analysis (7), list of upregulated and downregulated genes, and (8) conclusion. The mined details are listed in **Table 1**.

**Table 1:** Summary of selected studies.

Title (References)	Study design	Sampling	Sample size	Gene expression analysis	Upregulated genes	Downregulated genes
Expression of apoptosis-related genes in the endometrium of polycystic ovary syndrome patients during the window of implantation (14)	Case-control	Endometrium	Microarray (n = 6 PCOS, 6 control) PCR (n = 2 PCOS, 1 control)	Microarray: Affymetrix Oligo gene chips	88	73
Transcriptional profiling with a pathway-oriented analysis identifies dysregulated molecular phenotypes in the endometrium of patients with polycystic ovary syndrome (15)	Case-control	Endometrium	Microarray (n = 12 PCOS, 12 control) qPCR (n = 12 PCOS, 12 control)	Microarray: AffymetrixHumanGenomeU133A2.0 Genechip qPCR: DNA Engine Opticon 2 fluorescence detection system	50	50
Microarray evaluation of endometrial receptivity in Chinese women with polycystic ovary syndrome (10)	Case-control	Endometrium	Microarray (n = 9 PCOS, 7 control) qPCR (n = 9 PCOS, 7 control)	Microarray: Bio oligonucleotide microarrays qPCR: Mx3005P_TM_ Real Time PCR System	10	91

### Study Quality

The quality of each study was assessed independently by two authors (ZS and MHE) using the Joanna Briggs Institute critical appraisal tools for cross-sectional studies (16). The results of the quality assessment were validated by the other two authors (MAA and MFA). The studies were classified as low quality (high risk of bias) if the overall score was less than 50%, moderate quality (moderate risk of bias) if the overall score was 50–69%, and high quality (low risk of bias) if the overall score was more than 69%.

### Gene Ontology and Pathway Enrichment Analysis

Pooled DEGs were gathered from the studies that met the inclusion and exclusion criteria. All genes were analyzed using the Database for Annotation, Visualization, and Integrated Discovery (DAVID) to determine the cluster of genes that displayed significant functional-annotation enrichment related to PCOS’s pathogenesis (17, 18). The contribution of genes in the pathway related to PCOS was identified based on the Kyoto Encyclopedia of Genes and Genomes (KEGG) pathway, Biological Biochemical Image database (BBID), BIOCARTA pathway database, and Reactome.

### Protein–Protein Interaction Network, Clustering, and Visualization

The PPI network of DEGs were analyzed and clustered through PPI functional-enrichment analysis *via* STRING (PPI Functional-enrichment analysis) (https://string-db.org/) (19). To visualize molecular interaction networks and integrate gene-expression profiles of the DEGs, results from STRING were exported into Cytoscape software (http://www.cytoscape.org/) (20). Clusters of protein interaction that were highly related to the etiopathology of PCOS were also identified using Cytoscape. The Cytoscape MCODE plug-in was adopted to perform module analysis of the target network and protein clustering. The module-selection criteria included degree cutoff = 2, node score cutoff = 0.2, node density cutoff = 0.1, K-score = 2, and max depth = 100. The list of genes in the clusters was subsequently analyzed separately using STRING to identify significantly enriched ontology terms.

## Results

A total of 138 potentially relevant titles, published between the years 2001 to 2021, were identified from the four databases. EndNote X9 software by Clarivate Analytics (Philadelphia, PA, USA) was used as the reference manager. From the titles, 28 articles were identified as duplicates, and 110 articles were retrieved for abstract reviewing. Upon screening titles and abstracts, 36 articles were removed, resulting in the selection of 74 potentially pertinent articles for full-text review. Then, the full texts were thoroughly reviewed, and 71 articles were eliminated based on the inclusion and exclusion criteria. Finally, three articles were included in the present systematic review. All three studies were original research articles published between the years 2008 to 2012. Homogeneity of the selected studies was ensured by adhering to the specified inclusion and exclusion criteria to prevent sampling bias. Notably, all studies performed microarray for gene-expression analysis. Sample sizes for each study varied from nine to twelve PCOS samples. The characteristics of these studies are highlighted in **Table 1**.

### Study Quality

Detailed quality assessment of the included studies is shown in the **Supporting Material 1**. All of the included studies were of high-quality (low-risk of bias), with score of 80 to 100%.

### Identification of DEGs in the Endometrium of PCOS Women During the Implantation Window

**Figure 2:**
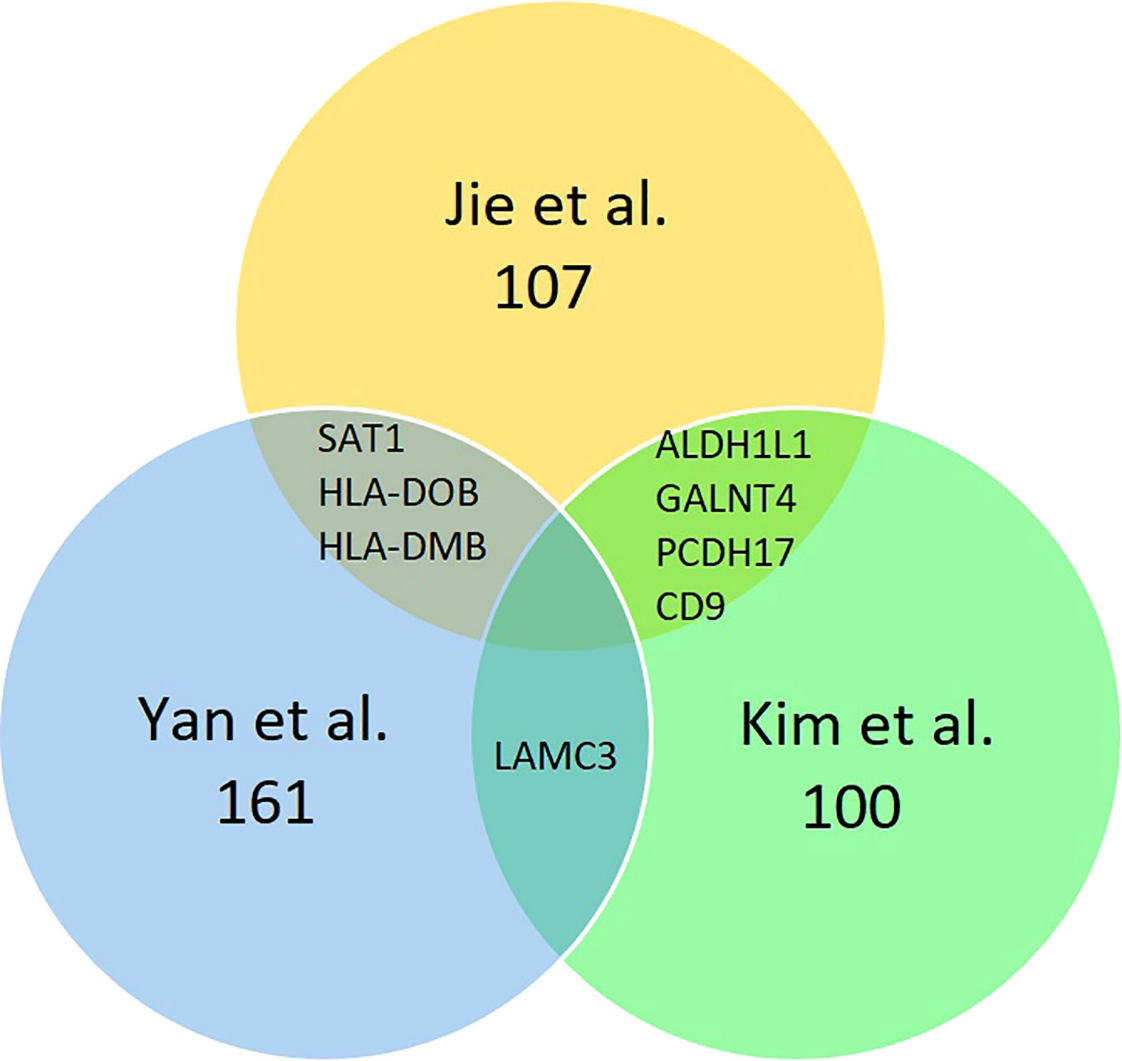
Identification of 368 DEGs from the three cohort datasets. Different color areas represent other datasets. The overlapping areas are the common differentially expressed genes.

In the studies of Yan et al. (14) and Jie et al. (10), only genes with the expression level of ≥2 or ≤−2 are identified as DEGs. However, Jie et al. (10) further specified the selection criteria by selecting genes that appeared only in at least five out of six PCOS women into their dataset. Meanwhile, Kim et al. (15) selected only the top 50 most increased and decreased genes into their dataset. A list of 161, 100, and 107 DEGs were extracted from the Yan et al. (14), Kim et al. (15), and Jie et al. (10) datasets, respectively. Kim et al. (15) shared a common DEG (LAMC3) with Yan et al. (14) and four common DEGs (CD9, PCDH17, ALDH1L1, CD9 and GALNT4) with Jie et al. (10), while Jie et al. (10) shared three common DEGs (SAT1, HLA-DOB and HLA-DMB) with Yan et al. (14). **Figure 2** summarise the DEGs distribution among the three studies. From the total of 368 DEGs extracted from the three studies, 154 genes were upregulated, and 214 genes were downregulated in PCOS women’ endometrium compared with those in normal women. A list of upregulated and downregulated genes is included in **Supporting Material 2**. Samples collections were described in detail in all studies. Yan et al. (14) collected endometrial samples from 12 PCOS women age 28 ± 3.5 years and 12 women with regular menstrual cycles aged 27 ± 3.4 years. Kim et al. (15) also collected endometrial samples from 12 normal cycling women and 12 patients with PCOS. Jie et al. (10) collected endometrial tissues from 9 women diagnosed with PCOS aged 31.67 ± 2.06 years and seven normal women aged 28.58 ± 4.54 years. Rotterdam consensus criteria were used to diagnose PCOS women in all the studies. The exclusion criteria included cryptorrhea, severe disease complications (14), other potential causes of anovulation or hyperandrogenemia (15), pregnancy, history of endometriosis, hypertension, diabetes mellitus, and other genetic disorders (10). Before endometrium biopsy, follicular development was monitored by transvaginal ultrasound and urine LH test from the 8th day of menstruation (mid-follicular phase) to determine the ovulation day. An endometrial biopsy was obtained during the mid-luteal phase in all studies.

### GO Analysis of the DEGs in the Endometrium of PCOS Women During Implantation Window.

A total of 368 DEGs in the endometrium of PCOS women were extracted from the three studies. The functional annotation of these genes, through GO were analyzed using DAVID (https://david.ncifcrf.gov/home.jsp). A p-value of <0.05 was used as a cutoff value. The genes listed were categorized into three functional-annotation categories of GO that included cellular component (CC), molecular function (MF), and biological process (BP). In the CC group, the downstream genes are enriched in the intracellular component of cells, including cytosol, nucleus, cytoplasm, plasma membrane, nucleoplasm, and membrane. The molecular function of DEGs is enriched in the protein binding, identical protein binding, ATP binding, and protein homodimerization activity. The biological process of DEGs is enriched in the signal transduction, positive regulation of transcription from RNA polymerase II promoter and apoptotic process. The complete list of the DEGs’ GOs is included in **Supporting Material 3**.

### Identification of Key Candidate Genes and Pathways in the PPI and Modular Analysis of the Downstream Genes

**Figure 3:**
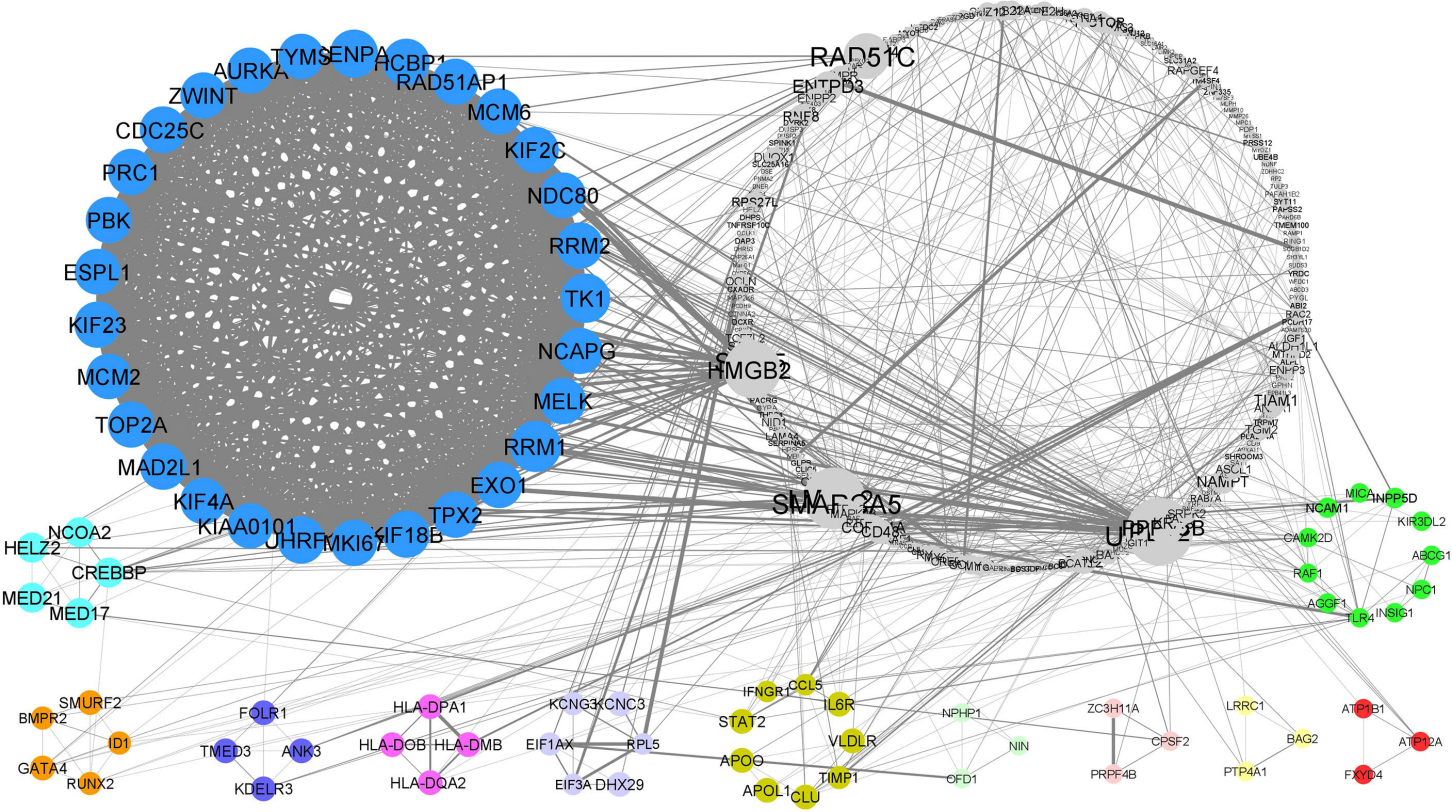
PPI network and modular analysis of downstream genes. From STRING online database analysis, a total of 283 proteins were filtered into a PPI network complex. Twelve clusters were identified from Cytoscape MCODE.

Using STRING online database (http://string-db.org), a total of 368 proteins from DEGs were filtered into a PPI network complex, containing 339 nodes and 1007 edges with a PPI enrichment p-value of <1.0e-16. The results were transferred from STRING to Cytoscape for visualizing the molecular interaction networks. Through Cytoscape MCODE, twelve significant modules from the PPI network complex were found. **Figure 3** shows the PPI network complex generated from the DEGs in the endometrium of PCOS women during the implantation window.

Functional-annotation clustering showed that cluster 1 (score = 27.643) comprised 29 nodes and 387 edges. The DEGs in cluster 1 were mostly located at intracellular and nucleus. The genes in cluster 1 involved in molecular binding, cell cycle, pyrimidine metabolism and oocyte meiosis. Cluster 2 (score= 5) is comprising of 5 nodes and 10 edges. The DEGs in cluster 2 work as transcription coactivator and are associated with thyroid hormone signalling pathway. Cluster 3 (score = 4.5), comprising 5 nodes and 9 edges, was associated with transcription regulation and TGF-beta signaling pathway. Cluster 4 (score = 4), comprising 4 nodes and 6 edges, was associated with the endoplasmic reticulum to golgi vesicle-mediated transport. Cluster 5 (score = 4), comprising 4 nodes and 6 edges, was associated with adaptive immune response. Cluster 6 (score = 3.6), comprising 6 nodes and 9 edges, was associated with the translational processes. Cluster 7 (score = 3), comprising 9 nodes and 12 edges, was associated with defence response and lipid transportation involving HIF- signaling pathway and Jak-STAT signaling pathway. Cluster 8, 9 and 10 (score = 3 respectively), comprising 3 nodes and 3 edges, showed no significant GOs. Cluster 11 (score =3), comprising 3 nodes and 3 edges, is associated with the cellular sodium and potassium ion homeostasis *via* aldosterone-regulated sodium reabsorption. Lastly, cluster 12 (score=2.6), comprising 11 nodes and 13 edges, is associated with immune system and cellular response to lipoprotein particle stimulus. **Table 2** shows the list of upregulated and downregulated genes according to their cluster. **Table 3** shows the list of significant functional annotations of all DEGs in each cluster. The full list of significant functional annotations of all DEGs in each cluster is included in **Supporting Material 4**.

**Table 2:** Clustering details of DEGs in endometrium of PCOS women during implantation window.

Cluster	Score	Nodes	Edges	Upregulated genes	Downregulated genes
**1**	27.64	29	387	–	AURKA, CDC25C, CENPA, ESPL1, EXO1, KIAA0101, KIF18B, KIF23, KIF2C, KIF4A, MAD2L1, MCM2, MCM6, MELK, MKI67, NCAPG, NDC80, PBK, PRC1, RAD51AP1, RRM1, RRM2, SHCBP1, TK1, TOP2A, TPX2, TYMS, UHRF1, ZWINT
**2**	5	5	10	MED21	CREBBP, HELZ2, MED17, NCOA2
**3**	4.5	5	9	SMURF2	BMPR2, GATA4, ID1, RUNX2,
**4**	4	4	6	–	ANK3, FOLR1, KDELR3, TMED3
**5**	4	4	6	HLA-DMB, HLA-DOB	HLA-DMB, HLA-DOB, HLA-DPA1, HLA-DQA2
**6**	3.6	6	9	DHX29, EIF1AX, EIF3A, KCNG3	KCNC3, RPL5
**7**	3	9	12	IL6R, STAT2, TIMP1, VLDLR	APOL1, APOO, CCL5, CLU, IFNGR1
**8**	3	3	3	NIN, NPHP1,	OFD1
**9**	3	3	3	CPSF2, PRPF4B, ZC3H11A	–
**10**	3	3	3	PTP4A1	BAG2, LRRC1
**11**	3	3	3	FXYD4	ATP12A, ATP1B1,
**12**	2.6	11	13	CAMK2D, INPP5D, INSIG1, MICA, RAF1, TLR4	ABCG1, AGGF1, KIR3DL2, NCAM1, NPC1,

**Table 3:** Functional annotation clustering on the clusters identified from DEGs.

Cluster	Term	Description	Count	p-value
1	CC_GO:0005622	Intracellular	29	0.0054
	CC_GO:0043229	Intracellular organelle	27	0.0155
	CC_GO:0005634	Nucleus	26	2.08E-06
	MF_GO:0005488	Binding	27	0.0363
	MF_GO:0005515	Protein binding	22	0.0032
	MF_GO:0003824	Catalytic activity	18	0.0203
	BP_GO:0007049	Cell cycle	26	1.54E-23
	BP_GO:0000278	Mitotic cell cycle	22	1.43E-22
	BP_GO:0016043	Cellular component organization	22	5.98E-05
	hsa04110	Cell cycle	5	0.0003
	hsa00240	Pyrimidine metabolism	4	0.0003
	hsa04114	Oocyte meiosis	4	0.0036
2	MF_GO:0003713	Transcription coactivator activity	5	3.73E-06
	MF_GO:0030374	Nuclear receptor transcription coactivator activity	3	0.0003
	MF_GO:0061629	RNA polymerase II-specific DNA-binding transcription factor binding	3	0.0196
	BP_GO:0045944	Positive regulation of transcription by rna polymerase ii	5	0.014
	hsa04919	Thyroid hormone signaling pathway	3	0.0008
3	BP_GO:0022603	Regulation of anatomical structure morphogenesis	5	0.001
	BP_GO:2000026	Regulation of multicellular organismal development	5	0.013
	BP_GO:0006357	Regulation of transcription by rna polymerase ii	5	0.013
	hsa04350	TGF-beta signaling pathway	3	0.0004
4	CC_GO:0005794	Golgi apparatus	4	0.0092
	CC_GO:0030660	Golgi-associated vesicle membrane	3	0.0012
	CC_GO:0030662	Coated vesicle membrane	3	0.0025
	BP_GO:0006888	Endoplasmic reticulum to golgi vesicle-mediated transport	4	0.00013
5	CC_GO:0042613	MHC class II protein complex	4	8.51E-10
	CC_GO:0005765	Lysosomal membrane	4	7.68E-05
	CC_GO:0010008	Endosome membrane	4	0.00012
	MF_GO:0032395	MHC class II receptor activity	3	2.28E-06
	MF_GO:0023026	MHC class II protein complex binding	2	0.0052
	BP_GO:0019886	Antigen processing and presentation of exogenous peptide antigen *via* mhc class ii	4	8.25E-06
	BP_GO:0002250	Adaptive immune response	4	0.00011
	hsa05310	Asthma	4	1.73E-09
	hsa04672	Intestinal immune network for IgA production	4	2.03E-09
	hsa04940	Type I diabetes mellitus	4	2.03E-09
6	CC_GO:0016282	Eukaryotic 43s preinitiation complex	2	0.0227
	MF_GO:0003743	Translation initiation factor activity	3	0.0012
	MF_GO:0005251	Delayed rectifier potassium channel activity	2	0.025
	BP_GO:0065003	Protein-containing complex assembly	5	0.0137
	BP_GO:0006413	Translational initiation	4	0.00055
	BP_GO:0006417	Regulation of translation	4	0.0118
7	CC_GO:0005615	Extracellular space	7	0.02
	CC_GO:0034358	Plasma lipoprotein particle	4	3.89E-06
	CC_GO:0034361	Very-low-density lipoprotein particle	3	5.03E-05
	BP_GO:0006952	Defense response	7	0.0023
	BP_GO:0009605	Response to external stimulus	7	0.0162
	BP_GO:0051246	Regulation of protein metabolic process	7	0.0276
	hsa04066	HIF-1 signaling pathway	3	0.0046
	hsa04630	JAK-STAT signaling pathway	3	0.0077
	hsa05164	Influenza A	3	0.0077
8,9,10	–	No significant GO found.	–	–
11	BP_GO:0006883	Cellular sodium ion homeostasis	2	0.008
	BP_GO:0030007	Cellular potassium ion homeostasis	2	0.008
	BP_GO:0036376	Sodium ion export across plasma membrane	2	0.008
	hsa04960	Aldosterone-regulated sodium reabsorption	2	0.0039
12	CC_GO:0009897	External side of plasma membrane	4	0.0429
	BP_GO:0071402	Cellular response to lipoprotein particle stimulus	3	0.0092
	hsa04650	Natural killer cell mediated cytotoxicity	3	0.0132
	hsa05152	Tuberculosis	3	0.0172
	hsa05205	Proteoglycans in cancer	3	0.018

## Discussion

The identification of the comprehensive molecular mechanisms of PCOS is essential to solve the infertility problem among PCOS women. Accordingly, numerous clinical studies have been conducted on gene expression to understand the mechanism underlying PCOS among infertile women (21–23). In this systematic review, we attempted to expand our understanding of the contribution of gene expression in the endometrium of PCOS women during the implantation window. From the analysis, twelve clusters were revealed from the pooled DEG dataset by using integrated bioinformatic analysis. The first cluster (cluster 1) contains 29 nodes, and 391 edges have a markedly high score of 27.64. A high number of edges in cluster 1 indicates high intermolecular interactions between the nodes (20). This finding shows that the DEGs in cluster 1 are highly connected and interacted, making them worthy of further study.

The genes in cluster 1 are all downregulated in PCOS endometrial cells during the implantation window. Their proteins are primarily located in the intracellular region, including the nucleus (GO:0005634), nucleoplasm (GO:0005654) and cytosol (GO:0005829). In the nucleus, some proteins of DEGs in cluster 1 reside in the centrosome (GO:0005813), mitotic spindle (GO:0072686) and chromosome (GO:0005694). Meanwhile, in the cytoplasm, the proteins of DEGs in cluster 1 reside in midbody (GO:0030496), various types of microtubules (GO:0015630; GO:0005874), and kinesin complex (GO:0005871). The location of the proteins in cluster 1 reveals that these proteins play roles in regulating the cell structure, especially during cell division. Hence, the downregulation of genes in cluster 1 could impair the regulation of cell division through their cell’s cytoskeleton.

In agreement with the protein locations, the functional-annotation enrichment analysis shows that genes in cluster 1 play an essential role in cell cycle (GO:0007049), cell cycle process (GO:0022402), cellular component organization (GO:0016043), cell division (GO:0051301), and DNA replication (GO:0006260). More specific to mitosis, some of the proteins in cluster 1 are involved in mitotic cell cycle (GO:0000278), mitotic nuclear division (GO:0140014), sister chromatid segregation (GO:0000819), and spindle organization (GO:0007051).

In normally ovulating women, decidualization starts with an acute stress response in endometrial stromal cells (EnSC) that results in cell cycle arrest. However, the presence of a discrete population of highly proliferating mesenchymal cells (hPMC) in the endometrium during implantation window was recently reported based on single-cell transcriptomics analysis. In this study, 127 upregulated genes were identified and from the GO, the genes are involved in cell cycle progression (24). Thus, in PCOS women, the downregulation of genes in cluster 1 could be one of the mechanisms that cause a reduction in endometrium cell cycle due to the lack of hPMC, which is a decidual precursor cell. The reduction in the endometrial cell cycle may interfere the decidualization process during implantation window.

From the systematic review and integrated bioinformatics analysis, five candidate genes were identified from cluster 1. Aurora Kinase A (AURKA), Cell division cycle 25C (CDC25C), Kinesin family member 23 (KIF23), Kinesin family member 2C (KIF2C), and NDC80 kinetochore complex component (NDC80) were selected as the candidate genes based on their significant involvement in biological processes. The GO analysis of these genes results in seven to ten biological processes. AURKA is a cell cycle-regulated kinase involved in microtubule formation and spindle-pole stabilization during chromosome segregation. It is found at the centrosome during interphase and at the spindle poles during mitosis (25). At the molecular level, the transcription factor C/EBPβ plays an essential role in human endometrial stromal cells decidualization. Interestingly, the interdependence between C/EBPβ and progesterone-receptor binding and the progesterone-receptor regulatory region of the AURKA gene has been reported (26). Hence, AURKA downregulation interferes with the cell-cycle processes of endometrial stomal cells. However, the effect of progesterone on AURKA expression and decidualization during the implantation window remains unknown. Hence, further studies on the decidualization mechanism *via* pathways involving AURKA are suggested.

CDC25C is a conserved protein that is important in regulating cell division. It directs the dephosphorylation of cyclin B-bound CDC2 and triggers entry into mitosis (27). From the GO analysis, CDC25C is distributed in the nucleus, nucleoplasm, and cytosol. The GO and pathway analysis show the involvement of CDC25C in DNA replication, cell division, cell proliferation, mitotic nuclear division, cell cycle, G2/M transition of mitotic cell cycle, and oocyte meiosis. In uterine stromal cells, loss of CDC25C expression contributes to G2 to M arrest of the cell cycle during decidualization (28). CDC25C are important in the successful progression of stromal cell decidualization in the endometrium during the implantation window (29). The decidualization process changes the function and morphology of the endometrium cells into the decidual lining, which is vital for blastocyst implantation (6). Hence, the downregulation of CDC25C in PCOS women could inhibit the decidualization process by inhibiting the cell cycle and leading to infertility. Meanwhile, PCOS women are mostly anovulatory and their progesterone levels are low during the implantation window (30, 31). Therefore, future studies on the effect of progesterone level toward CDC25C expression level during the implantation window in relation to decidualization progression among PCOS women are beneficial.

KIF23 and KIF2C are members of the kinesin-like protein family that are microtubule-dependent molecular motors involved in transporting organelles within cells. During mitosis, kinesin can also bind chromosomes to spindle fibers (32). From the GO analysis, KIF23 involves seven biological processes: mitotic cytokinesis, mitotic spindle elongation, meiotic spindle organization, and mitotic spindle midzone assembly. Conversely, KIF2C involves 10 biological processes: cell division, cell proliferation, sister chromatid cohesion, and mitotic nuclear division. KIF23 and KIF2C are involved in microtubule-based movement and retrograde vesicle-mediated transport from the Golgi apparatus to the endoplasmic reticulum. Hence, the downregulation of KIF23 and KIF2C reduces mitosis and cell-division activities in endometrium cells. Despite their significant involvement in cytokinesis, studies on the effect of these genes in the endometrium decidualization process during the implantation window are lacking. Thus, further studies on KIF23 and KIF2C in the mechanism of endometrium decidualization among PCOS women are also warranted.

NDC80 is important in organizing and stabilizing microtubule-kinetochore interactions and is required for proper chromosome segregation (33). GO reveals the involvement of NDC80 in cell division, mitotic sister chromatid segregation, sister chromatid cohesion, mitotic nuclear division, and positive regulation of mitotic cell-cycle spindle-assembly checkpoint. Hence, NCD80 downregulation in the endometrium of PCOS women during the implantation window may decrease cell cycle and cell division. In contrast to PCOS, NDC80 is reportedly overexpressed in endometrium cancer (34). However, further studies on the effect of NCD80 on endometrium decidualization among PCOS women are essential as related studies are lacking.

The main limiting factor in this review is the initial filtering and quality assessment of studies owing to different study structures, the incomplete listing of DEGs, and various statistical procedures used by the studies. However, the homogeneity of the data is assured by applying strict inclusion criteria. Selecting only microarray results for analysis could avoid bias in the selection of genes list. Unfortunately, the retrieved data from the studies did not include raw data of normal endometrium. Hence, the activation or deactivation of specific genes is unascertained. To improve the current knowledge, further functional studies of genes in cluster 1 are needed to elucidate the downstream mechanism in PCOS pathogenesis. RNA interference or gene-knockdown strategies can be used to explore the effect of these genes on the pathways and pathogenesis of PCOS. Nevertheless, this review added new insight into the PCOS molecular mechanism for future studies.

## Conclusions

Changes in the endometrium at the molecular level during the implantation window of PCOS women require investigation to identify the best solution for their infertility problems. From this systematic review and integrated bioinformatics analysis, cell cycle is the fundamental biological processes that is dysregulated in the endometrium of PCOS women during the implantation window. The cell cycle suppression of endometrial decidual precursor cell affects decidualization progression during the implantation window and are reflected by the changes in the endometrial gene expression. Thus, additional research is needed to further understand the influence of these genes on endometrial receptivity that lead to the poor reproductive performance among PCOS women. These future studies are important to identify potential biomarkers in predicting successful pregnancy rate among PCOS women undergo assisted reproductive technique (ART). In ART, like *In vitro* fertilization (IVF), identifying the optimal endometrial environment for implantation can help in the decision of embryo transfer among couples with limited number of quality embryos.

## Author Contributions

Article screening, selection, and data extraction, ZS, ME, MA, AK, MA; bioinformatic analysis, ME; writing—original draft preparation, ZS; writing—review and editing, ME, MA; visualization, MA, AK; supervision, MA; funding acquisition, MA. All authors have read and agreed to the published version of the manuscript.

## Funding

This research was funded by Fundamental Research Grant Scheme, awarded by Ministry of Higher Education of Malaysia, grant number FRGS/1/2018/SKK08/UKM/03/2. The authors have no competing interests to declare that are relevant to the content of this article.

## Conflict of Interest

The authors declare that the research was conducted in the absence of any commercial or financial relationships that could be construed as a potential conflict of interest.

## Publisher’s Note

All claims expressed in this article are solely those of the authors and do not necessarily represent those of their affiliated organizations, or those of the publisher, the editors and the reviewers. Any product that may be evaluated in this article, or claim that may be made by its manufacturer, is not guaranteed or endorsed by the publisher.
