# Supplementary material for: A Systematic Review and Integrated Bioinformatic Analysis of Candidate Genes and Pathways in the Endometrium of Patients With Polycystic Ovary Syndrome During the Implantation Window
Source: Front Endocrinol (Lausanne). 2022 Jul 1;13:900767. doi: 10.3389/fendo.2022.900767 (PMC9289743; doi:10.3389/fendo.2022.900767)
Supplement: Supplementary file 1 [file Table_1.docx]

| **Supplementary Table 1. Quality assessment of the included case control studies** | | | | | | | | | | | | |
| --- | --- | --- | --- | --- | --- | --- | --- | --- | --- | --- | --- | --- |
| **No.** | **Author, Year** | **Questions assessing – case control studies** | | | | | | | | | | **Yes (%)** |
|  |  | **1** | **2** | **3** | **4** | **5** | **6** | **7** | **8** | **9** | **10** |  |
| 1 | Jie et al., 2008 | Y | Y | Y | Y | Y | Y | Y | Y | Y | Y | 100 |
| 2 | Kim et al., 2009 | Y | Y | Y | Y | Y | Y | Y | Y | Y | Y | 100 |
| 3 | Yan et al., 2012 | Y | Y | Y | Y | Y | N | N | Y | Y | Y | 80 |
| Questions:  1. Were the groups comparable other than the presence of disease in cases or the absence of disease in controls?  2. Were cases and controls matched appropriately?  3. Were the same criteria used for identification of cases and controls?  4. Was exposure measured in a standard, valid and reliable way?  5. Was exposure measured in the same way for cases and controls?  6. Were confounding factors identified?  7. Were strategies to deal with confounding factors stated?  8. Were outcomes assessed in a standard, valid and reliable way for cases and controls?  9. Was the exposure period of interest long enough to be meaningful?  10.Was appropriate statistical analysis used?  Y=Yes; N=No; U=Unclear. | | | | | | | | | | | | |
