# Supplementary material for: A Systematic Review and Integrated Bioinformatic Analysis of Candidate Genes and Pathways in the Endometrium of Patients With Polycystic Ovary Syndrome During the Implantation Window
Source: Front Endocrinol (Lausanne). 2022 Jul 1;13:900767. doi: 10.3389/fendo.2022.900767 (PMC9289743; doi:10.3389/fendo.2022.900767)
Supplement: Supplementary file 2 [file Table_2.docx]

**Supp 2.** The 368 DEGs were identified from three datasets including 154 upregulated genes and 214 downregulated genes in the endometrium of PCOS women compared to endometrium of normal women.

| **Expression** | **Differentially expressed genes** |  |
| --- | --- | --- |
| **Upregulated** | SPIN1, MAP3K20, KMT2E, PARD6B, PPP1CB, CHTF8, CSNK1G1, CDKN2AIP, MAPK13, PRKX, SUZ12, MIB1, TLR4, UBE2H, SLC31A2, FXYD4, SAT1, TRPM7, KCNG3, CLIC5, ITGB2, MTSS1, SHROOM3, ABI2, GPHN, DLC1, NPHP1, BCL2, MOAP1, DIDO1, DAP3, TM2D1, SMURF2, NAMPT, EIF4G3, CPSF2, RPS27L, KPNA1, CAND1, RAD51C, BCS1L, HLA-DOB, RAB28, PRPF4B, RP2, RAPGEF4, MYO6, DYRK2, CAMK2D, IL6R, IGHM, AZGP1, VSIG2, CGA, HLA-DMB, RAB7A, KRAS, RAB21, RAB2A, GDI1, VAV3, RAF1, TRPM6, PIGW, MMP10, INSIG1, LIMK2, ENPP3, DCLK1, NIN, PLK2, PTP4A1, ZDHHC2, DHX29, EIF1AX, EIF3A , PTPRB, PPP6C, DUSP3, AAK1, SRPK2, IGFBP5, INPP5D, XRCC4, SCUBE3, TULP3, MED21, CNP, PACRG, TMEM100, LOC201229, CCDC6, NDRG2, MAP2K6, HMGA2, KAZALD1, ENPP2, ASCL1, STAT2, SOSTDC1, SLC25A16, ZNF335, FRAS1, PDP1, THBS4, MYOT, NSG1, LAMC3, FLJ11539, SATB1, PAX8, KIAA0663, FZD6, ASIC2, PLA1A, SEMA3A, VLDLR, OCLN, PCDH9, TRH, ALDH1L1, CD9, KIAA15336, BCAT1, MLPH, GALNT4, SH3YL1, CA11, MICA, FLJ44451, C1orf115, TIAM1, CRYL1, PRSS12, BSPRY, FLJ23191, CHD7, EST, PSPHP1, SNCA, HTRA3, ASS1, SMC5, TGM2, LOC144997, DHRS3, DNER, TIMP1, SMC5, FAF2, PCDH17, PSPHP1, KCND3, KIAA0040. |  |
| **Downregulated** | CUL2, GATA4, FABP3, UHRF1, UBE4B, RNF8, SUZ12, RING1, GLRB, RYR2, SCNN1G, KCNJ12, KCNC3, TPTE, LNPEP, ERAP2, GIT1, EPB41L3, CORO1A, CALD1, MYOZ1, RICTOR, LAMC3, BAX, BOK, FADD, TNFRSF10C, HLA-DPA1, HLA-DQA2, RNASE2, REXO2, RAG1, TATDN3, CHD2, HELZ, YTHDC2, HELZ2, MCM6, RUNX2, KIR3DL2, NCOA2, TCF7L2, HIC2, MBL2, CD48, GYPA, SPINK1, ARSK, PTPRG, LAIR2, OLFML2A, NCAM1, PTPN11, RAC2, BMP2K, GALT, MAN2B1, SEPSECS, SLC7A6, GDF11, DUSP13, TRAF1, SLC6A1, CREBBP, MUSK, MED17, SMARCA5, ADAMTS20, PAFAH1B2, CLU, BMPR2, CCL5, AGGF1, MTHFD2, LMNB2, RAMP1, SHCBP1, MELK, PAPSS2, TYMS, RPL5, BAG2, NCAPG, PNMA2, PRC1, TPX2, PBK, HMGB2, EXO1, TUBB6, PCDH17, LAMA4, RAD51AP1, MKI67, KIF2C, TOP2A, KIF18B, DSE, MAD2L1, KDELR3, KIF4A, AURKA, UBE2S, TK1, PCLAF, RRM2, ESPL1, COL5A2, COQ2, NDC80, FLNA, EXT1, RRM1, MCM2, MYL9, WFDC1, ZWINT, CENPA, SYT11, KIF23, CDC25C, OSTM1, NID1, TM4F4, ENTPD3, ATP12A, TFPI2, CTNNA2, MMP26, MUC15, SLC26A2, PKP2, SCGB1D2, ANK3, HPSE, KRT23, CYB5A, KRT80, EVA1A, SCGB2A2, ANXA1, PLA2G4A, ITGB4, ALPL, TRPM6, IFNGR1, CXADR, CD9, BST2, KRT8P12, TMED3, TM7SF3, OPRPN, NFE2, GMPR, DNAJC15, HSD11B2, CREB3L1, DUSP2, HPGD, RAB27B, PPM1H, GALNT4, HLA-DOB, HLA-DMB, SLC16A1, ATP1B1, IDH1, SERPINA5, OFD1, VTCN1, TPBG, FOLR1, MTARC1, SLC25A38, LRRC1, PDK4, P4HA1, DUOX1, APOO, DCXR, DHPS, ID1, XYLT2, SETD3, COQ7, IGF1, MAP1, ABCG1, ABCD3, YRDC, MPC1, LOC51581, FOXN1, SUDS3, PYGL, HRH1, Y11918, ALDH1L1, LOC51700, LDH3B2, CYP26A1, ANXA11, APOL1, RNF207, OPRPN, WFDC2, SNHG5, NPC1, KYAT1, SAT1, IMMP2L, GLIS3-AS1, GATAD1. |  |
